# Glomerular Filtration Rate as a Predictor of Outcome in Acute Coronary Syndrome Complicated by Atrial Fibrillation

**DOI:** 10.3390/jcm9051466

**Published:** 2020-05-14

**Authors:** Domenico Santoro, Guido Gembillo, Giuseppe Andò

**Affiliations:** 1 Unit of Nephrology and Dialysis, Department of Clinical and Experimental Medicine, University of Messina, 98125 Messina, Italy; dsantoro@unime.it (D.S.); guidogembillo@live.it (G.G.); 2 Unit of Cardiology, Department of Clinical and Experimental Medicine, University of Messina, 98125 Messina, Italy

**Keywords:** chronic kidney disease, glomerular filtration rate, atrial fibrillation, acute myocardial infarction, acute coronary syndrome

## Abstract

The close relationship between kidney and heart is well known. Cardiovascular impairment contributes to the worsening of renal function and kidney failure worsens cardiovascular health. Atrial fibrillation (AF) is a frequent issue in patients with Chronic Kidney Disease (CKD) and several studies have demonstrated that AF impacts negatively on their quality of life and outcomes. Understanding the mechanisms leading to the progression of CKD, new-onset AF and acute myocardial infarction (AMI) is a key issue. The evaluation of Glomerular Filtration Rate (GFR) could make the difference in this equilibrium and suggests specific strategies in the treatment of the population at major risk of cardiovascular events. This intriguing connection paves the way for necessary further investigations.

## 1. The Established Link between Kidney and Heart and the Role of Atrial Fibrillation (AF)

The close relationship between kidney and heart is well known. Cardiovascular impairment contributes to worsening of renal function and kidney failure worsens cardiovascular health. Atrial fibrillation (AF) is a frequent issue in Chronic Kidney Disease (CKD) patients, and several studies have demonstrated that this condition impacts negatively on their quality of life. AF, in turn, is associated with an increased risk of Acute Myocardial Infarction (AMI), having similar long-term prognosis despite these two conditions having a quite different pathogenesis [1,2].

The reason can be explained by mechanisms leading to CKD and AF and their pathophysiology. One of the altered pathways, common in both diseases, involves the renin–angiotensin–aldosterone system (RAAS). RAAS dysregulation is widely associated with hypertension and CKD progression and also to AF [3]. While angiotensin II can increase atrial pressure, with a three-fold increase in chronic persistent AF [4], treatment with RAAS inhibitors—on the other hand—may improve AF symptoms and reduce its incidence [5]. 

Moreover, a genetic link has been demonstrated between angiotensin-converting enzyme (ACE) polymorphisms and AF: ACE polymorphisms are associated with an increased risk of AF, together with vasoconstriction, an increased secretion of aldosterone and antidiuretic hormone, fibrosis and the structural remodeling of the atrial myocardium. These mechanisms facilitate the induction and maintenance of AF [6]. 

AF operates on heart pro-fibrotic effects and on the decline of left ventricular systolic and diastolic function [7,8], and these processes lead to hemodynamics perturbation. Indeed, AF contributes to the worsening of renal function because increased heart rate, an irregular sequence of ventricular cycles and the loss of atrial contribution to left ventricular filling lead to decreased cardiac output and, ultimately, to reduced kidney perfusion [9]. 

Incident AF in patients with CKD is also independently associated with an increased risk of developing End-Stage Renal Disease (ESRD) [10]. Moreover, in the international Dialysis Outcomes and Practice Patterns Study (DOPPS) on >17,000 dialysis patients, at study enrollment, AF was positively linked to all-cause mortality and stroke [11].

The reason could be partially found in the systemic inflammation and increased oxidative stress caused by AF that contributes to the development and progression of CKD [12,13]. CKD, also in early stages, is strongly linked with inflammation status [14], which is a bridge between AF and AMI as well, beside prothrombotic risk, systemic platelet activation, thrombin production and endothelial dysfunction. Such mechanisms stimulated by AF lead to AMI manifestations [15,16]. 

## 2. Impact of Acute Cardiac Conditions on Glomerular Filtration Rate (GFR) and the Role of Chronic Kidney Disease (CKD)

AMI directly affects the glomerular filtration rate (GFR), with a marked and rapid decrease after the cardiac event [17]. Other acute cardiological conditions having overlapping clinical presentation with AMI because of acutely reduced left ventricular function [18,19] may lead to acute kidney injury (AKI) as well [20]. More generally, about one quarter of patients suffering from acute coronary syndrome (ACS) develop AKI, and AKI after ACS is robustly correlated to in-hospital mortality [21]. Moreover, when AMI is complicated by cardiogenic shock, AKI exceeds 50% of cases [22]. CKD is also an established risk factor for AKI related to AMI; CKD patients have a much higher risk of AKI than subjects with normal renal function. This observation particularly applies to patients receiving contrast media during percutaneous intervention in the setting of AMI [23,24]. Taken all together, these data demonstrate that patients with combined AMI and CKD should be evaluated very carefully in clinical practice, since they represent a more vulnerable population [25]. 

Even for AMI, the RAAS pathway is a crucial mechanism, establishing a link between heart and kidney. As mentioned for AF and CKD, ACE inhibitors (ACE-I) play more important roles beyond being a fundamental first-line antihypertensive drug. ACE-I could also represent a protective factor against AKI after AMI. A clinical study of >6000 patients showed that RAAS inhibition, in patients with CKD and ACS, could improve 90-day mortality rates [26]. This is partially explained by the increased expression of serum level angiotensin II in subjects with AMI and AKI [27]. This evidence indicates a further close connection between CKD, AF, and AMI. The Niigata preventive medicine study confirmed that CKD is significantly associated with the risk of new-onset AF; AF, in turn, is shown to increase the risk of the development of CKD, creating a bivalent connection [28].

In line with these results, Cosentino and colleagues [29] performed a prospective observational study showing that kidney function was directly related to new-onset AF and had a relationship with short-term and long-term mortality in their cohort of AMI patients. They demonstrated a major incidence and risk of AF proportional to GFR declines, even after adjustment for major clinical predictors. AF patients had a significant increase in hospital mortality compared to non-AF subjects, at every GFR level. 

These evidences are supported by a recent systematic review and meta-analysis that demonstrated AF to be associated with an increased risk of death and cardiovascular and renal morbidity: AF patients had a 64% higher risk of CKD and a 96% higher risk of a major cardiovascular event [30].

## 3. Conclusions

Understanding the mechanisms leading to CKD progression, AF, and AMI is a key issue. Cardiovascular diseases are the main cause of death in CKD patients and the combination of AMI and AF may become a life threatening condition. The study by Cosentino and colleagues [29] provides, for the first time, specific data regarding different stages of renal function as predictors of AF in AMI. GFR evaluation could make the difference in this equilibrium and suggests specific strategies in the treatment of the population at a major risk of cardiovascular events. These findings accurately underline the importance of this connection, paving the way for necessary further investigations.

## References

[1] Behar S., Haim M., Hod H., Kornowski R., Reicher-Reiss H., Zion M., Kaplinsky E., Abinader E., Palant A., Kishon Y. (1996). Long-term prognosis of patients after a Q wave compared with a non-Q wave first acute myocardial infarction. Data from the SPRINT Registry. Eur. Heart J..

[2] Armstrong P.W., Fu Y., Chang W.C., Topol E.J., Granger C.B., Betriu A., Van de Werf F., Lee K.L., Califf R.M. (1998). Acute coronary syndromes in the GUSTO-IIb trial: Prognostic insights and impact of recurrent ischemia. The GUSTO-IIb Investigators. Circulation.

[3] Kobori H., Nangaku M., Navar L.G., Nishiyama A. (2007). The intrarenal renin-angiotensin system: From physiology to the pathobiology of hypertension and kidney disease. Pharm. Rev..

[4] Goette A., Staack T., Rocken C., Arndt M., Geller J.C., Huth C., Ansorge S., Klein H.U., Lendeckel U. (2000). Increased expression of extracellular signal-regulated kinase and angiotensin-converting enzyme in human atria during atrial fibrillation. J. Am. Coll. Cardiol..

[5] Pedersen O.D., Bagger H., Kober L., Torp-Pedersen C. (1999). Trandolapril reduces the incidence of atrial fibrillation after acute myocardial infarction in patients with left ventricular dysfunction. Circulation.

[6] Ravn L.S., Benn M., Nordestgaard B.G., Sethi A.A., Agerholm-Larsen B., Jensen G.B., Tybjaerg-Hansen A. (2008). Angiotensinogen and ACE gene polymorphisms and risk of atrial fibrillation in the general population. Pharm. Genom..

[7] Burstein B., Qi X.Y., Yeh Y.H., Calderone A., Nattel S. (2007). Atrial cardiomyocyte tachycardia alters cardiac fibroblast function: A novel consideration in atrial remodeling. Cardiovasc. Res..

[8] Naito M., David D., Michelson E.L., Schaffenburg M., Dreifus L.S. (1983). The hemodynamic consequences of cardiac arrhythmias: Evaluation of the relative roles of abnormal atrioventricular sequencing, irregularity of ventricular rhythm and atrial fibrillation in a canine model. Am. Heart J..

[9] Clark D.M., Plumb V.J., Epstein A.E., Kay G.N. (1997). Hemodynamic effects of an irregular sequence of ventricular cycle lengths during atrial fibrillation. J. Am. Coll. Cardiol..

[10] Bansal N., Fan D., Hsu C.Y., Ordonez J.D., Marcus G.M., Go A.S. (2013). Incident atrial fibrillation and risk of end-stage renal disease in adults with chronic kidney disease. Circulation.

[11] Wizemann V., Tong L., Satayathum S., Disney A., Akiba T., Fissell R.B., Kerr P.G., Young E.W., Robinson B.M. (2010). Atrial fibrillation in hemodialysis patients: Clinical features and associations with anticoagulant therapy. Kidney Int..

[12] Chung M.K., Martin D.O., Sprecher D., Wazni O., Kanderian A., Carnes C.A., Bauer J.A., Tchou P.J., Niebauer M.J., Natale A. (2001). C-reactive protein elevation in patients with atrial arrhythmias: Inflammatory mechanisms and persistence of atrial fibrillation. Circulation.

[13] Pena J.M., MacFadyen J., Glynn R.J., Ridker P.M. (2012). High-sensitivity C-reactive protein, statin therapy, and risks of atrial fibrillation: An exploratory analysis of the JUPITER trial. Eur. Heart J..

[14] Landray M.J., Wheeler D.C., Lip G.Y., Newman D.J., Blann A.D., McGlynn F.J., Ball S., Townend J.N., Baigent C. (2004). Inflammation, endothelial dysfunction, and platelet activation in patients with chronic kidney disease: The chronic renal impairment in Birmingham (CRIB) study. Am. J. Kidney Dis..

[15] Skalidis E.I., Zacharis E.A., Tsetis D.K., Pagonidis K., Chlouverakis G., Yarmenitis S., Hamilos M., Manios E.G., Vardas P.E. (2007). Endothelial cell function during atrial fibrillation and after restoration of sinus rhythm. Am. J. Cardiol..

[16] Wong C.X., Lim H.S., Schultz C.D., Sanders P., Worthley M.I., Willoughby S.R. (2012). Assessment of endothelial function in atrial fibrillation: Utility of peripheral arterial tonometry. Clin. Exp. Pharmacol. Physiol..

[17] Mashima Y., Konta T., Ichikawa K., Ikeda A., Suzuki K., Wanezaki M., Nishiyama S., Watanabe T., Kubota I. (2013). Rapid decline in renal function after acute myocardial infarction. Clin. Nephrol..

[18] Ando G., Trio O., de Gregorio C. (2010). Transient left ventricular dysfunction in patients with neurovascular events. Acute Card Care.

[19] Trio O., de Gregorio C., Ando G. (2010). Myocardial dysfunction after subarachnoid haemorrhage and tako-tsubo cardiomyopathy: A differential diagnosis?. Ther. Adv. Cardiovasc. Dis..

[20] Yassin A.S., Adegbala O., Subahi A., Abubakar H., Akintoye E., Abdelrahamn M., Ahmed A., Agarwal A., Shokr M., Pahuja M. (2019). Clinical impact of advanced chronic kidney disease on outcomes and in-hospital complications of Takotsubo Syndrome (broken-heart-syndrome): Propensity-matched national study. Int. J. Cardiol..

[21] Hwang S.H., Jeong M.H., Ahmed K., Kim M.C., Cho K.H., Lee M.G., Ko J.S., Park K.H., Sim D.S., Yoon N.S. (2011). Different clinical outcomes of acute kidney injury according to acute kidney injury network criteria in patients between ST elevation and non-ST elevation myocardial infarction. Int. J. Cardiol..

[22] Marenzi G., Assanelli E., Campodonico J., De Metrio M., Lauri G., Marana I., Moltrasio M., Rubino M., Veglia F., Montorsi P. (2010). Acute kidney injury in ST-segment elevation acute myocardial infarction complicated by cardiogenic shock at admission. Crit. Care Med..

[23] Ando G., Morabito G., de Gregorio C., Trio O., Saporito F., Oreto G. (2013). Age, glomerular filtration rate, ejection fraction, and the AGEF score predict contrast-induced nephropathy in patients with acute myocardial infarction undergoing primary percutaneous coronary intervention. Catheter. Cardiovasc. Interv..

[24] Ando G., Morabito G., de Gregorio C., Trio O., Saporito F., Oreto G. (2013). The ACEF score as predictor of acute kidney injury in patients undergoing primary percutaneous coronary intervention. Int. J. Cardiol..

[25] Wang C., Pei Y.Y., Ma Y.H., Ma X.L., Liu Z.W., Zhu J.H., Li C.S. (2019). Risk factors for acute kidney injury in patients with acute myocardial infarction. Chin. Med. J..

[26] Reddan D.N., Szczech L., Bhapkar M.V., Moliterno D.J., Califf R.M., Ohman E.M., Berger P.B., Hochman J.S., Van de Werf F., Harrington R.A. (2005). Renal function, concomitant medication use and outcomes following acute coronary syndromes. Nephrol. Dial. Transplant..

[27] Liu K.L., Lee K.T., Chang C.H., Chen Y.C., Lin S.M., Chu P.H. (2014). Elevated plasma thrombomodulin and angiopoietin-2 predict the development of acute kidney injury in patients with acute myocardial infarction. Crit. Care.

[28] Watanabe H., Watanabe T., Sasaki S., Nagai K., Roden D.M., Aizawa Y. (2009). Close bidirectional relationship between chronic kidney disease and atrial fibrillation: The Niigata preventive medicine study. Am. Heart J..

[29] Cosentino N., Ballarotto M., Campodonico J., Milazzo V., Bonomi A., Genovesi S., Moltrasio M., De Metrio M., Rubino M., Veglia F. (2020). Impact of Glomerular Filtration Rate on the Incidence and Prognosis of New-Onset Atrial Fibrillation in Acute Myocardial Infarction. J. Clin. Med..

[30] Odutayo A., Wong C.X., Hsiao A.J., Hopewell S., Altman D.G., Emdin C.A. (2016). Atrial fibrillation and risks of cardiovascular disease, renal disease, and death: Systematic review and meta-analysis. BMJ.

